# The Reliability and Validity of the Persian Version of Sinonasal Outcome Test 22 (Snot 22) Questionnaires

**DOI:** 10.5812/ircmj.7937

**Published:** 2013-05-05

**Authors:** Maryam Jalessi, Mohammad Farhadi, Seyyed Kamran Kamrava, Ebrahim Amintehran, Alimohammad Asghari, Mohsen Rezaei Hemami, Alireza Mobasseri, Mohammadreza Masroorchehr

**Affiliations:** 1 ENT-Head and Neck Research Center and Department, Hazrat Rasoul Akram Hospital, Tehran University of Medical Sciences, Tehran, IR Iran; 2 Department of Iranian Traditional Medicine, School of Traditional Medicine, Shahid Beheshti University of Medical Sciences, Tehran, IR Iran

**Keywords:** Quality of Life, Nasal Septal, Questionnaires

## Abstract

**Background:**

The quality of life (QOL) is an important indicator for disease-severity classification and outcome measurement in obtaining treatment sinonasal diseases. The sinonasal outcome test 22 (SNOT 22) questionnaire has been introduced as the best specific sinonasal instrument for QOL measurement.

**Objectives:**

To prepare a valid and reliable Persian language version of SNOT 22 questionnaire.

**Patients and Methods:**

After forward and backward translation of the original version of SNOT 22 questionnaire from English to Persian, a group of patients with nasal septal deviation who need septal surgery and another group of healthy volunteers answered the Persian version of the questionnaire. The responsiveness rate, validity (Pearson correlations and differential validity) and reliability (internal consistency and test-retest reliability) of the 22 items of the questionnaire was calculated. P value < 0.05 was considered significant.

**Results:**

Thirty adults with nasal septal deviation need surgical correction and 30 healthy volunteers were included (mean age 30.4 ± 7.1 vs. 33 ± 6.7, P value = 0.148). The questionnaire was introduced to subjects two times with a two-week-period gap. Total responsiveness rate for 22 items was more than 97%. The total Cronbach's Alfa coefficient was 0.898 (ranging 0.890-0.903). The Pearson correlations were 0.85 and 0.96 for patients and healthy volunteers, respectively. The mean total score were 25.6 ± 13.3 (range 6-52) and 7.6 ± 9.1 (range 0-45) in patients and healthy volunteers, respectively (P < 0.0001). The subscales scores were also significantly different between two groups.

**Conclusions:**

The Persian version of SNOT 22 questionnaire is a valid and reliable instrument for accessing sinonasal diseases in Persian-speaking people.

## 1. Background

Measuring the quality of life (QOL) of patients with sinonasal diseases is a useful indicator for disease- severity classification, best treatment obtaining, and the outcome evaluating. There are some generic and specific instruments for assessing the QOL in patients with sinonasal diseases (1). The generic instruments measure the QOL of patients with different diseases and are capable of comparing the QOL among them. The specific instruments have the most-relating items to a specific disease, making them a better choice for treatment evaluation (2). Among the specific instruments, the SNOT 22 questionnaire has been introduced as the best one for determining the QOL in sinonasal diseases (3). The SNOT 22 questionnaire which was developed in 2003 but validated in 2009, has been used in a variety of sinonasal diseases (e.g. chronic rhino-sinusitis (CRS), nasal septal deviation, Wegener granulomatosis, Churg-Strauss syndrome) successfully (1, 4-7). This questionnaire contains 22 items, covering the most relevant symptoms of sinonasal diseases. Each item is graded in 6 levels (0 for no problem, 5 for the problem as bad as it can be). The final score is gained by adding the score of each item (range 0-110), the greater the score, the worse quality of life. The SNOT 22 questionnaire is the advanced prototype of previous version, the SNOT 20, which lacks the two items of nasal blockage and changes of taste and smell. The questionnaire has the advantages of being filled easily and quickly by the patients. It also gains an estimation of quality of life of the patients by simply calculating the total score. As SNOT-22 is a self-reporting questionnaire, it has been translated and validated in some other languages (8-12).

## 2. Objectives

The aim of our study was to prepare a valid and reliable Persian language version of the SNOT22 questionnaire for further use in Persian-speaking people.

## 3. Patients and Methods

### 3.1. Translation

An early Persian version of the questionnaire was prepared by two official English to Persian translators separately. The translators were instructed to prepare a context for general population, avoiding medical terms and exact word to word translation. Then these two early drafts were evaluated by four ENT specialists. They revised the early draft and prepared one for backward translation by another translator from Persian to English. Concerning this backward translated draft, the final draft was then prepared and used in study sample. Appendix 1 represents the Persian language version of the questionnaire.

### 3.2. Patients

A group of patients with nasal septal deviation candidate for septoplasty were enrolled in the study. The study was conducted from March to June 2012 in a tertiary ENT center in Tehran, Iran. Only adults older than 18 years old were included. The diagnosis of nasal septal deviation was made by otorhinolaryngologist based on clinical examination, anterior rhinoscopy, and coronal CT scans. A group of healthy volunteers were also included. They were adults with no history of chronic sinonasal disease. They were not taking any medication related to sinonasal problems. The people in this group were selected from the family members of the patients or personnel of the center. Both groups answered the final draft of the Persian version of SNOT 22. All participants in both groups were asked to answer the questionnaire again in a two-week period. The patients group re-answered the questionnaire before any surgical intervention.

### 3.3. Statistics

The internal consistency was determined for assessing the reliability of the Persian version of the questionnaire. The Cronbach's Alfa coefficient was calculated for all items at first and then by removing each item at once. Test-retest reliability was also calculated as another measure of reliability by paired t-test and determining the Pearson correlation between the two experiments. The validity was measured in two ways: adding all items to make a total score and then determining the relation of total score item with other 22 items (Pearson correlation), and comparing the total scored among two groups (differential validity). The responsiveness rate of the questionnaire was determined as the feasibility capacity of the questionnaire, showing how many of participants were able to answer the items by their own. Statistical analysis was performed using SPSS version 19 software (SPSS Inc., Chicago, IL, USA). P value < 0.05 was considered significant.

### 3.4. Ethical Views

The study protocol was proved by the ethical committee of the center. All participants agreed to take part in the study, free to withdraw in any step.

## 4. Results

There were 30 patients (18 females, 12 males) and 30 subjects (19 females, 11 males) in patients and healthy volunteers groups, respectively. Healthy volunteers were slightly older than patients (mean age 33 ± 6.7 vs. 30.4 ± 7.1, P > 0.05). Table 1 demonstrates general characteristics of participants.

**Table 1.** General Characteristics of Patients and Healthy Volunteers

	Patients with Nasal Septal Deviation (n = 30)	Healthy Volunteers (n = 30)	P value
**Gender**			0.79
Female	18	19	
Male	12	11	
**Age, y, Mean ± SD**	30.4 ± 7.1	33 ± 6.7	0.148

The feasibility of Persian version was accessed by the number of responded items. Total responsiveness of this version for 22 items was more than 97% and 16 items were responded in all subjects. The total Cronbach's Alfa coefficient for 22 items was 0.898, ranging 0.890-0.903 when removing one item at once. This coefficient indicates a good internal consistency (homogeneity among different items). For test-retest reliability all patients and healthy volunteers refilled the questionnaires in a period of two weeks from the first enrollment. The Pearson correlations were 0.85 and 0.96 for patients and healthy volunteers, respectively. By considering these results, the reliability of Persian version of the questionnaire was passed. Validity of the Persian version of the questionnaire was determined by Pearson correlation. The Pearson correlations more than 0.5 with P value less than 0.05 were considered as items with good significant correlations with other items in the questionnaire. Table 2 shows the Pearson correlation for each item.

**Table 2.** Cronbach

Item No.	Item in English Language Version	Cronbach's Alfa Coefficient	Pearson Correlation
**1**	Need to blow nose	0.56	0.62
**2**	Sneezing	0.56	0.61
**3**	Runny nose	0.50	0.57
**4**	Nasal obstruction	0.49	0.55
**5**	Loss of smell or taste	0.60	0.67
**6**	Cough	0.46	0.53
**7**	Post-nasal discharge	0.41	0.48
**8**	Thick nasal discharge	0.28	0.35
**9**	Ear fullness	0.44	0.48
**10**	Dizziness	0.42	0.46
**11**	Ear pain	0.61	0.67
**12**	Facial pain/pressure	0.57	0.63
**13**	Difficulty falling asleep	0.57	0.62
**14**	Waking up at night	0.66	0.71
**15**	Lack of a good night’s sleep	0.60	0.66
**16**	Waking up tired	0.44	0.50
**17**	Fatigue	0.38	0.44
**18**	Reduced productivity	064	0.70
**19**	Reduced concentration	0.44	0.49
**20**	Frustrated/restless/irritable	0.38	0.45
**21**	Sad	0.29	0.37
**22**	Embarrassed	0.74	0.80

The questionnaire must be able to differentiate patients with sinonasal diseases from healthy volunteers. The total score of 22 items is a good indicator of the group the subject belongs. The differentiate validity of this Persian version was accessed by comparing the total scores in both groups. The total score was 25.6± 13.3 (range 6-52) and 7.6± 9.1 (range 0-45) in patients and healthy volunteers groups, respectively (P < 0.0001).

**Table 3.** Total scores in two groups

	Mean ± SD	Range	P value
**Patients with nasal septal deviation**	47.6 ± 13.3	28-74	0.0001
**Healthy volunteers **	29.6 ± 9.1	22-67	

We also calculated the scores in subscales and compared them between two groups for further studies. Table 4 demonstrates the mean scores in 6 subscales according Lange et al. ( 9 ) and Browne et al. ( 13 ) in two groups.

**Table 4.** The Mean Subscales Scores in Two Groups

Subscales	Items No.	Patients with Nasal Septal Deviation (n = 30), Mean ± SD	Healthy Volunteers (n = 30), Mean ± SD	P value
**Physical symptoms**	1-12	14 ± 7.8	3.9 ± 6.3	0.001
**Psychological symptoms **	13-22	11.6 ± 8.4	3.7 ± 3.8	0.001
**Rhinologic symptoms (1, 13)**	1-3,7,8	6 ± 4.3	1.7 ± 2.9	0.001
**Rhinologic symptoms (2, 9)**	1-5,7,8	9.2 ± 5.6	2.5 ± 4	0.001
**Ear and facial symptoms**	9-12	3.4 ± 3.3	1.2 ± 2.1	0.004
**Sleep function**	13-15	4.2 ± 4.1	1.6 ± 2	0.003
**Psychological issues**	16-22	7.4 ± 5.3	2.1 ± 2.4	0.001

## 5. Discussion

Evaluating the quality of life of patients with sinonasal diseases is a key element in their treatment program. Instruments measuring this key factor also are valuable, if they are valid and reliable. The primary goal of our current study was to prepare a valid and reliable Persian language version of SNOT 22 questionnaire. As the instrument is a self-reporting questionnaire, the presence of any interviewer may cause bias in results and participants should be able to answer questions by themselves. For these reasons, this version of questionnaire was applied to all participants without any other explanation. In the translation step, we also tried to prepare a context understandable for general population. The responsiveness rate of 97% showed the intelligibility and feasibility of all items. The reliability is the ability of an instrument to produce constant results in constant situations. Stability is a key element in reliability, not influenced by time or individual's characteristics. Although there is no way for direct measuring of reliability, it could be estimated in some ways. The reliability of the questionnaire was tested in two ways: internal consistency and test-retest reliability. The Cronbach's Alfa coefficient of about 0.9 showed a good internal consistency. The test-retest reliability measures were 0.85 and 0.96 for patients and healthy volunteers respectively. These results were confirming the stability of the questionnaire over time. The validity is the accordance between the value measured and the one which is in the real world. Validity helps to truly measure what the observer claims to measure. Like reliability, there are some ways to estimate validity of instruments. In this study, the validity of this version of SNOT 22 questionnaire was tested by Pearson correlations and its ability to differentiate patients from healthy volunteers. The later was assessed by comparing the mean total scores between two groups. The average score in patients group was 25.6± 13.3 while the average score in healthy volunteers was 7.6 ± 9.1 (P < 0.001). In another study (4) performed on a group of patients preparing for septoplasty, the mean score was 36.3 preoperatively. Similar studies (8, 12) among CRS patients reported mean score of 64.54 and 38.52, respectively. The observed difference is probably due to different study samples, not a significant factor in interpretation of sinonasal tests. There was a similarity between the mean score of healthy volunteers in our study and previous investigations (8, 14). Like other studies, no relevant physical examination was carried out in this group. Although the diversity in healthy volunteers group in different studies may have a role, there is still a chance of presence of mild sinonasal conditions among healthy volunteers, mostly unaware of their disease. Another explanation of this situation could be due to that residents of metropolitan (like Tehran, the capital of Iran) may have a greater degree of sinonasal symptoms, as the overlapping score ranges between group of patients and healthy volunteers indicate. However, this theory needs to be evaluated in a big scale population. The mean scores were also calculated in 6 subscales according to previous studies (9, 13). Although there is no global agreement in defining subscales, it is clear that in any classification of items, the patients always reach higher scores comparing to healthy individuals. This is another verification of the questionnaire and this special classification of the symptoms. We also tried a new group of patients with nasal septal deviation which was different from other studies on chronic rhino-sinusitis patients. Our results showed that the questionnaire has the capacity of being used in other sinonasal patients population. One limitation of our study was that we did not introduce the questionnaire post-operatively. However, it has been well documented that the SNOT22 questionnaire is well capable of being used as a before-after questionnaire in patients undergoing sinonasal surgeries. Future studies could demonstrate this version's capability for other sinonasal disease evaluation pre and post-operatively. Further studies are required to approve other aspects of the SNOT 22 questionnaire. We prepared a valid and reliable version of the instrument for future use. Measuring the health-related quality of life in patients in ENT departments needs more thorough evaluation, considering symptoms relief and disease-free duration. The Persian version of SNOT 22 questionnaire is a valid and reliable instrument for being used in accessing quality of life in patients with sinonasal diseases in Persian-speaking individuals.
